# Crystal structure of hydro­cortisone 17-butyrate

**DOI:** 10.1107/S1600536814023903

**Published:** 2014-11-05

**Authors:** Yu Zhu, Wei Shen, Hai-li Wang

**Affiliations:** a College of Chemistry and Molecular Engineering, Zhengzhou University, Zhengzhou 450001, People’s Republic of China

**Keywords:** crystal structure, hydro­cortisone derivative, hydrogen bonds, pharmacological activity

## Abstract

In the title compound, C_25_H_36_O_6_, the two central cyclo­hexane rings exhibit a chair conformation. The terminal cyclo­hexene and cyclo­pentane rings are in half-chair and envelope conformations (with the C atom bearing the methyl substit­uent as the flap), respectively. The methyl group of the butyrate chain is disordered over two orientations, with a refined occupancy ratio of 0.742 (6):0.258 (6). Intra­molecular O—H⋯O and C—H⋯O hydrogen bonds are observed. In the crystal, mol­ecules are linked by O—H⋯O hydrogen bonds into chains running parallel to the *a* axis.

## Related literature   

For the pharmacological activities of the title compound, see: Haapasaari *et al.* (1995 ▶); Lerche *et al.* (2010 ▶); D’Erme & Gola (2012 ▶). For the synthesis of the title compound, see: Sun *et al.* (2009 ▶).
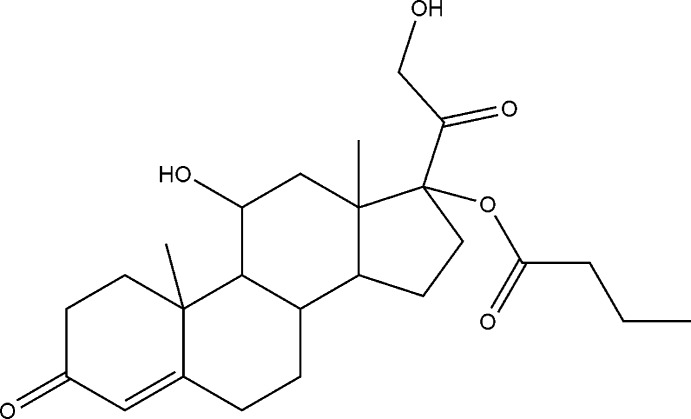


## Experimental   

### Crystal data  

C_25_H_36_O_6_
*M* *_r_* = 432.54
Orthorhombic, 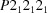

*a* = 9.05738 (8) Å
*b* = 11.87633 (9) Å
*c* = 21.13465 (15) Å
*V* = 2273.42 (3) Å^3^
*Z* = 4
Cu *K*α radiation
μ = 0.72 mm^−1^
*T* = 291 K
0.22 × 0.2 × 0.2 mm

### Data collection  

Agilent Xcalibur Eos Gemini diffractometer
Absorption correction: multi-scan (*CrysAlis PRO* (Agilent, 2011 ▶) *T*_min_ = 0.952, *T*_max_ = 1.000
23353 measured reflections
4496 independent reflections
4389 reflections with *I* > 2σ(*I*)
*R*_int_ = 0.021

### Refinement  

*R*[*F* ^2^ > 2σ(*F* ^2^)] = 0.040
*wR*(*F* ^2^) = 0.111
*S* = 1.06
4496 reflections
301 parameters
4 restraints
H atoms treated by a mixture of independent and constrained refinement
Δρ_max_ = 0.31 e Å^−3^
Δρ_min_ = −0.28 e Å^−3^
Absolute structure: Flack (1983 ▶), 1932 Friedel pairs
Absolute structure parameter: 0.02 (18)

### 

Data collection: *CrysAlis PRO* (Agilent, 2011 ▶); cell refinement: *CrysAlis PRO*; data reduction: *CrysAlis PRO*; program(s) used to solve structure: *SHELXS97* (Sheldrick, 2008 ▶); program(s) used to refine structure: *SHELXL97* (Sheldrick, 2008 ▶); molecular graphics: *OLEX2* (Dolomanov *et al.*, 2009 ▶); software used to prepare material for publication: *OLEX2*.

## Supplementary Material

Crystal structure: contains datablock(s) I, global. DOI: 10.1107/S1600536814023903/rz5136sup1.cif

Structure factors: contains datablock(s) I. DOI: 10.1107/S1600536814023903/rz5136Isup2.hkl

Supporting information file. DOI: 10.1107/S1600536814023903/rz5136Isup3.cml

**Fig. 1.**
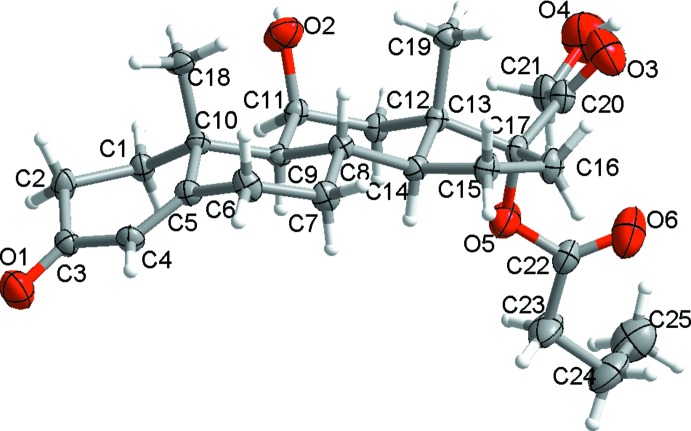
The molecular structure of the title compound showing 30% probability displacement ellipsoids. Only the major component of the disordered C25 methyl group is shown

. DOI: 10.1107/S1600536814023903/rz5136fig1.tif
The mol­ecular structure of the title compound showing 30% probability displacement ellipsoids. Only the major component of the disordered C25 methyl group is shown

CCDC reference: 1031721

Additional supporting information:  crystallographic information; 3D view; checkCIF report

## Figures and Tables

**Table 1:** Hydrogen-bond geometry (, )

*D*H*A*	*D*H	H*A*	*D* *A*	*D*H*A*
C19H19*C*O2	0.96	2.39	3.016(2)	122
O4H4O3	0.83(3)	2.06(3)	2.629(3)	126(2)
O2H2O1^i^	0.82	2.11	2.9192(18)	169

Symmetry code: (i) 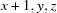.

## supplementary crystallographic information

### S1. Comment

Hydrocortisone 17-butyrate is an important cortical hormone drug derived from the esterification reaction of hydrocortisone at the hydroxyl group of C-17. Due to the introduction of the alkyl chain, hydrocortisone 17-butyrate showed increased lipophicity and affinity for receptors, which lead to increased pharmacological activity (Haapasaari *et al.*, 1995; Lerche *et al.*, 2010; D'Erme & Gola, 2012). Compared with hydrocortisone, it showed increased anti-inflammatory activity, immunosuppressive properties, and low side effect. Due to the outstanding characteristics of hydrocortisone 17-butyrate, it has drawn great attention of the experts from the fields of chemistry, pharmacy and medicine. The synthesis and properties have been investigated quite extensively, while its molecular structure has not been reported. Here we present the single-crystal X-ray diffraction study of hydrocortisone 17-butyrate.

The molecular structure of the title compound is shown in Figure 1. As expected, the chiral carbon atoms C8, C9, C11, C13, and C14 exhibit *S* configuration, and atoms C10 and C17 exhibit *R* configuration. The Flack parameter is 0.02 (18). Both central six membered rings (C5, C6, C7, C8, C9, C10, and C8, C9, C11, C12, C13, C14) exhibit chair conformation, with atoms C5 and C8 displaced by 0.6024 (16) and 0.6378 (15) Å on opposite sides from the C6, C7, C9 and C10 plane, and atoms C9 and C13 by 0.6249 (14) and 0.7014 (15) Å from the C8, C11, C12 and C14 plane. The cyclohexene ring (C1, C2, C3, C4, C5, C10) assumes a half-chair conformation, atom C2 protruding by 0.534 (2) Å from the mean plane through the remaining five atoms. The cyclopentane ring is in an envelope conformation, with atom C13 displaced by 0.6761 (15) Å from the C14, C15, C16, C17 mean plane. Intramolecular O—H···O and C—H···O hydrogen bonds are observed (Table 1). In the crystal, molecules are connected by intermolecular O—H···O hydrogen bonds to form chains parallel to the *a* axis.

### S2. Experimental

The title compound was obtained following a patent report (Sun *et al.*, 2009). At 0-5°C, butyryl chloride (1.5 mmol, 0.16 mL) was added dropwise to a CH_2_Cl_2_ (10 mL) solution containing hydrocortisone 21-acetate (1 mmol, 0.4 g), Et_3_N (4 mL) and 4-dimethylaminopyridine (0.05 mmol, 6 mg). The mixture was then stirred at 0°C for 3 hours before being treated with HCl to reach a pH of 2. The mixture was then washed with H_2_O to reach neutrality, and extracted using CH_2_Cl_2_. The organic phase was combined, dried, and evaporated. Crystallization of the residue in MeOH produced hydrocortisone 17-butyrate 21-acetate. The CH_2_Cl_2_ solution of hydrocortisone 17-butyrate 21-acetate was added slowly to a MeOH (10 mL) solution of K_2_CO_3_ (0.1 g) at -10°C for selective hydrolysis. The mixture was then neutralized by CH_3_COOH, washed with H_2_O, and extracted with CH_2_Cl_2_. The organic phase was combined, dried and evaporated. Crystallization of the residue in MeOH at 0°C produced pure hydrocortisone 17-butyrate. Crystals suitable for X-ray analysis were obtained by slow evaporation of a MeOH (20 mL) solution of hydrocortisone 17-butyrate (5 mg) at room temperature.

### S3. Refinement

The C25 methyl carbon atom is disordered over two orientations with refined occupancy ratio 0.742 (6):0.258 (6). The disordered atoms were refined by constraining the C24–C25 and C24–C25A bond lengths to be 1.52 (1) Å and by restraining the anisotropic displacement ellipsoids to be equal. The hydroxyl H atom bound to O4 was located in a difference Fourier map and refined freely. All other H atoms were placed in calculated positions and refined as riding, with C—H = 0.93–0.97 Å, O—H = 0.82 Å, and with *U*_iso_(H) = 1.2*U*_eq_(C) or 1.5*U*_eq_(C) for methyl and hydroxyl H atoms. A rotating model was applied to the methyl and hydroxyl groups. One outlier (0 1 1) was omitted in the last cycles of refinement.

### Crystal data

**Table:**

C_25_H_36_O_6_	*D*_x_ = 1.264 Mg m^−^^3^
*M**_r_* = 432.54	Cu *K*α radiation, λ = 1.54184 Å
Orthorhombic, *P*2_1_2_1_2_1_	Cell parameters from 14007 reflections
*a* = 9.05738 (8) Å	θ = 4.2–72.4°
*b* = 11.87633 (9) Å	µ = 0.72 mm^−^^1^
*c* = 21.13465 (15) Å	*T* = 291 K
*V* = 2273.42 (3) Å^3^	Block, colourless
*Z* = 4	0.22 × 0.2 × 0.2 mm
*F*(000) = 936	

### Data collection

**Table:**

Agilent Xcalibur Eos Gemini diffractometer	4496 independent reflections
Radiation source: Enhance (Cu) X-ray Source	4389 reflections with *I* > 2σ(*I*)
Graphite monochromator	*R*_int_ = 0.021
Detector resolution: 16.2312 pixels mm^-1^	θ_max_ = 72.3°, θ_min_ = 4.2°
ω scans	*h* = −9→11
Absorption correction: multi-scan (*CrysAlis PRO* (Agilent, 2011)	*k* = −14→14
*T*_min_ = 0.952, *T*_max_ = 1.000	*l* = −26→26
23353 measured reflections	

### Refinement

**Table:**

Refinement on *F*^2^	Secondary atom site location: difference Fourier map
Least-squares matrix: full	Hydrogen site location: inferred from neighbouring sites
*R*[*F*^2^ > 2σ(*F*^2^)] = 0.040	H atoms treated by a mixture of independent and constrained refinement
*wR*(*F*^2^) = 0.111	*w* = 1/[σ^2^(*F*_o_^2^) + (0.0712*P*)^2^ + 0.3126*P*] where *P* = (*F*_o_^2^ + 2*F*_c_^2^)/3
*S* = 1.06	(Δ/σ)_max_ = 0.001
4496 reflections	Δρ_max_ = 0.31 e Å^−^^3^
301 parameters	Δρ_min_ = −0.28 e Å^−^^3^
4 restraints	Absolute structure: Flack (1983), 1932 Friedel pairs
Primary atom site location: structure-invariant direct methods	Absolute structure parameter: 0.02 (18)

### Special details

**Table:**

Geometry. All e.s.d.'s (except the e.s.d. in the dihedral angle between two l.s. planes) are estimated using the full covariance matrix. The cell e.s.d.'s are taken into account individually in the estimation of e.s.d.'s in distances, angles and torsion angles; correlations between e.s.d.'s in cell parameters are only used when they are defined by crystal symmetry. An approximate (isotropic) treatment of cell e.s.d.'s is used for estimating e.s.d.'s involving l.s. planes.
Refinement. Refinement of *F*^2^ against ALL reflections. The weighted *R*-factor *wR* and goodness of fit *S* are based on *F*^2^, conventional *R*-factors *R* are based on *F*, with *F* set to zero for negative *F*^2^. The threshold expression of *F*^2^ > σ(*F*^2^) is used only for calculating *R*-factors(gt) *etc*. and is not relevant to the choice of reflections for refinement. *R*-factors based on *F*^2^ are statistically about twice as large as those based on *F*, and *R*- factors based on ALL data will be even larger.

### Fractional atomic coordinates and isotropic or equivalent isotropic displacement parameters (Å^2^)

**Table:**

	*x*	*y*	*z*	*U*_iso_*/*U*_eq_	Occ. (<1)
O1	−0.36156 (14)	0.73133 (12)	0.93301 (6)	0.0499 (3)	
O2	0.38389 (14)	0.61706 (12)	0.87774 (6)	0.0468 (3)	
H2	0.4512	0.6572	0.8906	0.070*	
O3	0.70484 (17)	0.51666 (13)	0.64786 (8)	0.0624 (4)	
O4	0.8532 (2)	0.7026 (2)	0.67034 (11)	0.0791 (5)	
H4	0.866 (3)	0.638 (2)	0.6577 (13)	0.059 (7)*	
O5	0.39480 (15)	0.69247 (10)	0.63513 (5)	0.0448 (3)	
O6	0.5163 (2)	0.66663 (14)	0.54407 (7)	0.0670 (4)	
C1	0.02943 (18)	0.72375 (15)	0.90894 (8)	0.0409 (3)	
H1A	0.1177	0.7472	0.9314	0.049*	
H1B	0.0142	0.7759	0.8743	0.049*	
C2	−0.1022 (2)	0.73130 (18)	0.95395 (9)	0.0483 (4)	
H2A	−0.0831	0.6860	0.9912	0.058*	
H2B	−0.1154	0.8087	0.9674	0.058*	
C3	−0.24009 (18)	0.69069 (14)	0.92219 (7)	0.0391 (3)	
C4	−0.22232 (18)	0.59526 (15)	0.87958 (8)	0.0408 (3)	
H4A	−0.3071	0.5621	0.8632	0.049*	
C5	−0.09161 (18)	0.55240 (13)	0.86262 (7)	0.0373 (3)	
C6	−0.0827 (2)	0.44526 (14)	0.82502 (9)	0.0453 (4)	
H6A	−0.1814	0.4225	0.8126	0.054*	
H6B	−0.0415	0.3862	0.8513	0.054*	
C7	0.0122 (2)	0.45906 (15)	0.76617 (9)	0.0440 (4)	
H7A	−0.0378	0.5087	0.7367	0.053*	
H7B	0.0237	0.3864	0.7458	0.053*	
C8	0.16479 (17)	0.50722 (13)	0.78101 (7)	0.0336 (3)	
H8	0.2217	0.4516	0.8051	0.040*	
C9	0.15146 (16)	0.61680 (12)	0.82020 (7)	0.0311 (3)	
H9	0.0957	0.6684	0.7930	0.037*	
C10	0.05504 (16)	0.60493 (14)	0.88151 (7)	0.0339 (3)	
C11	0.30159 (18)	0.67602 (13)	0.83072 (7)	0.0355 (3)	
H11	0.2813	0.7517	0.8471	0.043*	
C12	0.39077 (18)	0.68924 (12)	0.76887 (7)	0.0352 (3)	
H12A	0.3440	0.7461	0.7427	0.042*	
H12B	0.4893	0.7156	0.7791	0.042*	
C13	0.40305 (17)	0.57970 (12)	0.73084 (7)	0.0336 (3)	
C14	0.24614 (18)	0.53463 (12)	0.71963 (7)	0.0340 (3)	
H14	0.1906	0.5951	0.6989	0.041*	
C15	0.2669 (2)	0.44163 (14)	0.67029 (8)	0.0438 (4)	
H15A	0.1773	0.4307	0.6459	0.053*	
H15B	0.2934	0.3710	0.6903	0.053*	
C16	0.3930 (2)	0.48456 (16)	0.62797 (8)	0.0483 (4)	
H16A	0.3556	0.5039	0.5864	0.058*	
H16B	0.4679	0.4268	0.6232	0.058*	
C17	0.4592 (2)	0.58969 (13)	0.66039 (7)	0.0388 (3)	
C18	0.1214 (2)	0.52665 (18)	0.93282 (8)	0.0505 (4)	
H18A	0.0463	0.5073	0.9630	0.076*	
H18B	0.2007	0.5648	0.9540	0.076*	
H18C	0.1583	0.4593	0.9133	0.076*	
C19	0.5017 (2)	0.49307 (15)	0.76391 (8)	0.0423 (4)	
H19A	0.5989	0.5239	0.7693	0.063*	
H19B	0.5073	0.4261	0.7386	0.063*	
H19C	0.4607	0.4750	0.8045	0.063*	
C20	0.6275 (2)	0.59745 (15)	0.65638 (8)	0.0460 (4)	
C21	0.7000 (3)	0.7106 (2)	0.66925 (12)	0.0609 (5)	
H21A	0.672 (3)	0.740 (3)	0.7116 (14)	0.076 (8)*	
H21B	0.656 (4)	0.773 (3)	0.6395 (17)	0.100 (11)*	
C22	0.4291 (3)	0.71949 (17)	0.57472 (9)	0.0525 (5)	
C23	0.3459 (4)	0.8215 (2)	0.55363 (12)	0.0835 (8)	
H23A	0.2414	0.8036	0.5535	0.100*	
H23B	0.3612	0.8807	0.5846	0.100*	
C24	0.3861 (5)	0.8661 (3)	0.49010 (13)	0.0950 (10)	
H24A	0.3902	0.8041	0.4602	0.114*	0.742 (6)
H24B	0.3098	0.9176	0.4760	0.114*	0.742 (6)
H24C	0.4756	0.8270	0.4776	0.114*	0.258 (6)
H24D	0.3093	0.8410	0.4614	0.114*	0.258 (6)
C25	0.5263 (6)	0.9239 (4)	0.4900 (2)	0.1017 (15)	0.742 (6)
H25A	0.5190	0.9910	0.5151	0.153*	0.742 (6)
H25B	0.5522	0.9436	0.4474	0.153*	0.742 (6)
H25C	0.6008	0.8755	0.5074	0.153*	0.742 (6)
C25A	0.4109 (19)	0.9858 (11)	0.4760 (7)	0.1017 (15)	0.258 (6)
H25D	0.3263	1.0159	0.4545	0.153*	0.258 (6)
H25E	0.4965	0.9935	0.4496	0.153*	0.258 (6)
H25F	0.4262	1.0263	0.5148	0.153*	0.258 (6)

### Atomic displacement parameters (Å^2^)

**Table:**

	*U*^11^	*U*^22^	*U*^33^	*U*^12^	*U*^13^	*U*^23^
O1	0.0363 (6)	0.0593 (7)	0.0543 (7)	0.0030 (5)	0.0045 (5)	−0.0085 (6)
O2	0.0376 (6)	0.0639 (8)	0.0387 (6)	−0.0106 (6)	−0.0084 (5)	0.0032 (5)
O3	0.0531 (8)	0.0558 (8)	0.0784 (10)	0.0067 (6)	0.0191 (7)	−0.0034 (7)
O4	0.0565 (9)	0.0848 (13)	0.0961 (14)	−0.0210 (9)	0.0018 (9)	−0.0035 (11)
O5	0.0565 (7)	0.0433 (6)	0.0346 (5)	0.0032 (5)	0.0059 (5)	0.0017 (5)
O6	0.0899 (12)	0.0657 (9)	0.0454 (7)	−0.0036 (8)	0.0209 (8)	−0.0014 (7)
C1	0.0325 (7)	0.0491 (9)	0.0411 (8)	−0.0043 (6)	0.0009 (6)	−0.0120 (7)
C2	0.0396 (9)	0.0630 (11)	0.0424 (8)	−0.0048 (8)	0.0017 (7)	−0.0162 (8)
C3	0.0339 (8)	0.0467 (9)	0.0367 (7)	−0.0017 (6)	0.0030 (6)	0.0003 (6)
C4	0.0315 (7)	0.0467 (8)	0.0443 (8)	−0.0054 (7)	−0.0030 (6)	−0.0040 (7)
C5	0.0348 (8)	0.0376 (7)	0.0395 (7)	−0.0034 (6)	−0.0011 (6)	0.0012 (6)
C6	0.0357 (8)	0.0385 (8)	0.0618 (10)	−0.0092 (7)	0.0071 (7)	−0.0071 (8)
C7	0.0400 (9)	0.0414 (8)	0.0505 (9)	−0.0077 (7)	−0.0009 (7)	−0.0115 (7)
C8	0.0341 (7)	0.0305 (6)	0.0363 (7)	−0.0022 (6)	0.0002 (6)	−0.0014 (6)
C9	0.0317 (7)	0.0300 (6)	0.0317 (6)	0.0007 (5)	−0.0030 (5)	−0.0010 (5)
C10	0.0288 (7)	0.0407 (8)	0.0324 (7)	−0.0001 (6)	−0.0025 (5)	0.0013 (6)
C11	0.0371 (8)	0.0354 (7)	0.0340 (7)	−0.0057 (6)	0.0000 (6)	−0.0054 (6)
C12	0.0389 (8)	0.0298 (7)	0.0369 (7)	−0.0061 (6)	0.0028 (6)	−0.0044 (6)
C13	0.0364 (7)	0.0309 (7)	0.0336 (7)	−0.0027 (6)	0.0019 (6)	−0.0031 (6)
C14	0.0386 (8)	0.0302 (7)	0.0332 (7)	−0.0023 (6)	−0.0029 (6)	−0.0021 (5)
C15	0.0508 (9)	0.0395 (8)	0.0410 (8)	−0.0078 (7)	0.0012 (7)	−0.0106 (7)
C16	0.0569 (10)	0.0461 (9)	0.0418 (8)	−0.0085 (8)	0.0066 (8)	−0.0141 (7)
C17	0.0472 (9)	0.0338 (7)	0.0353 (7)	−0.0016 (7)	0.0050 (6)	−0.0047 (6)
C18	0.0401 (9)	0.0691 (12)	0.0422 (8)	0.0010 (8)	−0.0015 (7)	0.0187 (8)
C19	0.0439 (9)	0.0392 (8)	0.0438 (8)	0.0031 (7)	−0.0041 (7)	−0.0021 (7)
C20	0.0509 (10)	0.0455 (9)	0.0417 (8)	−0.0044 (8)	0.0107 (7)	−0.0035 (7)
C21	0.0530 (11)	0.0623 (12)	0.0675 (13)	−0.0153 (10)	0.0141 (10)	−0.0139 (10)
C22	0.0687 (12)	0.0512 (10)	0.0375 (8)	−0.0054 (9)	0.0054 (8)	−0.0015 (7)
C23	0.123 (2)	0.0740 (15)	0.0538 (12)	0.0197 (16)	0.0049 (14)	0.0188 (11)
C24	0.146 (3)	0.0853 (19)	0.0542 (13)	−0.007 (2)	−0.0056 (17)	0.0197 (12)
C25	0.112 (4)	0.098 (3)	0.096 (3)	−0.031 (3)	−0.003 (3)	0.017 (2)
C25A	0.112 (4)	0.098 (3)	0.096 (3)	−0.031 (3)	−0.003 (3)	0.017 (2)

### Geometric parameters (Å, º)

**Table:**

O1—C3	1.223 (2)	C12—C13	1.5332 (19)
O2—H2	0.8200	C13—C14	1.537 (2)
O2—C11	1.426 (2)	C13—C17	1.578 (2)
O3—C20	1.202 (2)	C13—C19	1.531 (2)
O4—H4	0.83 (3)	C14—H14	0.9800
O4—C21	1.391 (3)	C14—C15	1.531 (2)
O5—C17	1.454 (2)	C15—H15A	0.9700
O5—C22	1.353 (2)	C15—H15B	0.9700
O6—C22	1.199 (3)	C15—C16	1.538 (3)
C1—H1A	0.9700	C16—H16A	0.9700
C1—H1B	0.9700	C16—H16B	0.9700
C1—C2	1.528 (2)	C16—C17	1.545 (2)
C1—C10	1.543 (2)	C17—C20	1.530 (3)
C2—H2A	0.9700	C18—H18A	0.9600
C2—H2B	0.9700	C18—H18B	0.9600
C2—C3	1.497 (2)	C18—H18C	0.9600
C3—C4	1.457 (2)	C19—H19A	0.9600
C4—H4A	0.9300	C19—H19B	0.9600
C4—C5	1.338 (2)	C19—H19C	0.9600
C5—C6	1.502 (2)	C20—C21	1.520 (3)
C5—C10	1.521 (2)	C21—H21A	1.00 (3)
C6—H6A	0.9700	C21—H21B	1.05 (4)
C6—H6B	0.9700	C22—C23	1.495 (3)
C6—C7	1.521 (3)	C23—H23A	0.9700
C7—H7A	0.9700	C23—H23B	0.9700
C7—H7B	0.9700	C23—C24	1.488 (4)
C7—C8	1.528 (2)	C24—H24A	0.9700
C8—H8	0.9800	C24—H24B	0.9700
C8—C9	1.5473 (19)	C24—H24C	0.9700
C8—C14	1.527 (2)	C24—H24D	0.9700
C9—H9	0.9800	C24—C25	1.443 (6)
C9—C10	1.569 (2)	C24—C25A	1.470 (12)
C9—C11	1.547 (2)	C25—H25A	0.9600
C10—C18	1.550 (2)	C25—H25B	0.9600
C11—H11	0.9800	C25—H25C	0.9600
C11—C12	1.545 (2)	C25A—H25D	0.9600
C12—H12A	0.9700	C25A—H25E	0.9600
C12—H12B	0.9700	C25A—H25F	0.9600
			
C11—O2—H2	109.5	C14—C15—H15A	110.9
C21—O4—H4	101 (2)	C14—C15—H15B	110.9
C22—O5—C17	116.96 (14)	C14—C15—C16	104.36 (13)
H1A—C1—H1B	107.7	H15A—C15—H15B	108.9
C2—C1—H1A	108.8	C16—C15—H15A	110.9
C2—C1—H1B	108.8	C16—C15—H15B	110.9
C2—C1—C10	113.88 (15)	C15—C16—H16A	110.2
C10—C1—H1A	108.8	C15—C16—H16B	110.2
C10—C1—H1B	108.8	C15—C16—C17	107.35 (13)
C1—C2—H2A	109.5	H16A—C16—H16B	108.5
C1—C2—H2B	109.5	C17—C16—H16A	110.2
H2A—C2—H2B	108.1	C17—C16—H16B	110.2
C3—C2—C1	110.66 (13)	O5—C17—C13	106.28 (12)
C3—C2—H2A	109.5	O5—C17—C16	111.11 (14)
C3—C2—H2B	109.5	O5—C17—C20	109.19 (14)
O1—C3—C2	122.63 (15)	C16—C17—C13	103.46 (13)
O1—C3—C4	121.47 (15)	C20—C17—C13	112.19 (14)
C4—C3—C2	115.82 (15)	C20—C17—C16	114.24 (14)
C3—C4—H4A	118.0	C10—C18—H18A	109.5
C5—C4—C3	124.03 (15)	C10—C18—H18B	109.5
C5—C4—H4A	118.0	C10—C18—H18C	109.5
C4—C5—C6	120.77 (15)	H18A—C18—H18B	109.5
C4—C5—C10	123.13 (14)	H18A—C18—H18C	109.5
C6—C5—C10	116.07 (14)	H18B—C18—H18C	109.5
C5—C6—H6A	109.3	C13—C19—H19A	109.5
C5—C6—H6B	109.3	C13—C19—H19B	109.5
C5—C6—C7	111.82 (14)	C13—C19—H19C	109.5
H6A—C6—H6B	107.9	H19A—C19—H19B	109.5
C7—C6—H6A	109.3	H19A—C19—H19C	109.5
C7—C6—H6B	109.3	H19B—C19—H19C	109.5
C6—C7—H7A	109.1	O3—C20—C17	122.78 (17)
C6—C7—H7B	109.1	O3—C20—C21	118.72 (18)
C6—C7—C8	112.56 (14)	C21—C20—C17	118.30 (16)
H7A—C7—H7B	107.8	O4—C21—C20	112.0 (2)
C8—C7—H7A	109.1	O4—C21—H21A	105.3 (18)
C8—C7—H7B	109.1	O4—C21—H21B	115.8 (19)
C7—C8—H8	109.3	C20—C21—H21A	111.5 (17)
C7—C8—C9	110.74 (13)	C20—C21—H21B	111 (2)
C9—C8—H8	109.3	H21A—C21—H21B	101 (3)
C14—C8—C7	109.98 (13)	O5—C22—C23	110.96 (18)
C14—C8—H8	109.3	O6—C22—O5	122.50 (19)
C14—C8—C9	108.24 (11)	O6—C22—C23	126.5 (2)
C8—C9—H9	104.7	C22—C23—H23A	108.4
C8—C9—C10	114.20 (12)	C22—C23—H23B	108.4
C10—C9—H9	104.7	H23A—C23—H23B	107.4
C11—C9—C8	113.00 (12)	C24—C23—C22	115.7 (3)
C11—C9—H9	104.7	C24—C23—H23A	108.4
C11—C9—C10	114.29 (12)	C24—C23—H23B	108.4
C1—C10—C9	108.16 (12)	C23—C24—H24A	109.0
C1—C10—C18	110.12 (14)	C23—C24—H24B	109.0
C5—C10—C1	110.03 (13)	C23—C24—H24C	106.2
C5—C10—C9	107.82 (12)	C23—C24—H24D	106.2
C5—C10—C18	106.05 (13)	H24A—C24—H24B	107.8
C18—C10—C9	114.58 (13)	H24A—C24—H24C	55.2
O2—C11—C9	109.66 (12)	H24A—C24—H24D	52.2
O2—C11—H11	107.6	H24B—C24—H24C	144.4
O2—C11—C12	111.49 (13)	H24B—C24—H24D	59.4
C9—C11—H11	107.6	H24C—C24—H24D	106.4
C12—C11—C9	112.59 (12)	C25—C24—C23	112.7 (3)
C12—C11—H11	107.6	C25—C24—H24A	109.0
C11—C12—H12A	108.9	C25—C24—H24B	109.0
C11—C12—H12B	108.9	C25—C24—H24C	59.5
H12A—C12—H12B	107.7	C25—C24—H24D	140.9
C13—C12—C11	113.29 (12)	C25—C24—C25A	53.5 (7)
C13—C12—H12A	108.9	C25A—C24—C23	124.3 (7)
C13—C12—H12B	108.9	C25A—C24—H24A	126.6
C12—C13—C14	108.01 (12)	C25A—C24—H24B	55.7
C12—C13—C17	117.02 (12)	C25A—C24—H24C	106.2
C14—C13—C17	100.28 (12)	C25A—C24—H24D	106.2
C19—C13—C12	111.91 (13)	C24—C25—H25A	109.5
C19—C13—C14	112.07 (12)	C24—C25—H25B	109.5
C19—C13—C17	107.06 (13)	C24—C25—H25C	109.5
C8—C14—C13	112.92 (12)	C24—C25A—H25D	109.5
C8—C14—H14	106.7	C24—C25A—H25E	109.5
C8—C14—C15	118.96 (13)	C24—C25A—H25F	109.5
C13—C14—H14	106.7	H25D—C25A—H25E	109.5
C15—C14—C13	104.06 (13)	H25D—C25A—H25F	109.5
C15—C14—H14	106.7	H25E—C25A—H25F	109.5
			
O1—C3—C4—C5	175.19 (17)	C10—C9—C11—C12	−178.22 (13)
O2—C11—C12—C13	74.10 (17)	C11—C9—C10—C1	57.46 (16)
O3—C20—C21—O4	−1.1 (3)	C11—C9—C10—C5	176.41 (12)
O5—C17—C20—O3	−147.68 (18)	C11—C9—C10—C18	−65.79 (18)
O5—C17—C20—C21	37.6 (2)	C11—C12—C13—C14	54.22 (17)
O5—C22—C23—C24	174.0 (3)	C11—C12—C13—C17	166.34 (14)
O6—C22—C23—C24	−5.4 (5)	C11—C12—C13—C19	−69.61 (18)
C1—C2—C3—O1	−145.75 (18)	C12—C13—C14—C8	−61.36 (15)
C1—C2—C3—C4	37.2 (2)	C12—C13—C14—C15	168.27 (12)
C2—C1—C10—C5	42.57 (18)	C12—C13—C17—O5	−37.73 (19)
C2—C1—C10—C9	160.11 (14)	C12—C13—C17—C16	−154.84 (15)
C2—C1—C10—C18	−73.98 (18)	C12—C13—C17—C20	81.54 (18)
C2—C3—C4—C5	−7.8 (3)	C13—C14—C15—C16	−34.65 (17)
C3—C4—C5—C6	172.34 (16)	C13—C17—C20—O3	94.8 (2)
C3—C4—C5—C10	−5.4 (3)	C13—C17—C20—C21	−80.0 (2)
C4—C5—C6—C7	127.75 (18)	C14—C8—C9—C10	173.70 (12)
C4—C5—C10—C1	−12.2 (2)	C14—C8—C9—C11	−53.41 (16)
C4—C5—C10—C9	−130.00 (16)	C14—C13—C17—O5	78.71 (13)
C4—C5—C10—C18	106.83 (19)	C14—C13—C17—C16	−38.40 (15)
C5—C6—C7—C8	52.7 (2)	C14—C13—C17—C20	−162.02 (13)
C6—C5—C10—C1	169.87 (14)	C14—C15—C16—C17	9.6 (2)
C6—C5—C10—C9	52.12 (18)	C15—C16—C17—O5	−95.58 (17)
C6—C5—C10—C18	−71.05 (18)	C15—C16—C17—C13	18.09 (19)
C6—C7—C8—C9	−52.40 (19)	C15—C16—C17—C20	140.35 (17)
C6—C7—C8—C14	−171.99 (14)	C16—C17—C20—O3	−22.6 (3)
C7—C8—C9—C10	53.07 (17)	C16—C17—C20—C21	162.70 (18)
C7—C8—C9—C11	−174.04 (13)	C17—O5—C22—O6	−5.1 (3)
C7—C8—C14—C13	−177.98 (13)	C17—O5—C22—C23	175.4 (2)
C7—C8—C14—C15	−55.61 (19)	C17—C13—C14—C8	175.66 (12)
C8—C9—C10—C1	−170.26 (12)	C17—C13—C14—C15	45.28 (14)
C8—C9—C10—C5	−51.30 (16)	C17—C20—C21—O4	173.85 (19)
C8—C9—C10—C18	66.49 (17)	C19—C13—C14—C8	62.37 (16)
C8—C9—C11—O2	−75.82 (15)	C19—C13—C14—C15	−68.01 (16)
C8—C9—C11—C12	48.93 (17)	C19—C13—C17—O5	−164.22 (13)
C8—C14—C15—C16	−161.34 (15)	C19—C13—C17—C16	78.68 (16)
C9—C8—C14—C13	60.92 (15)	C19—C13—C17—C20	−44.94 (17)
C9—C8—C14—C15	−176.71 (14)	C22—O5—C17—C13	179.33 (15)
C9—C11—C12—C13	−49.64 (18)	C22—O5—C17—C16	−68.78 (19)
C10—C1—C2—C3	−55.8 (2)	C22—O5—C17—C20	58.11 (19)
C10—C5—C6—C7	−54.3 (2)	C22—C23—C24—C25	−74.3 (4)
C10—C9—C11—O2	57.03 (16)	C22—C23—C24—C25A	−134.6 (9)

### Hydrogen-bond geometry (Å, º)

**Table:**

*D*—H···*A*	*D*—H	H···*A*	*D*···*A*	*D*—H···*A*
C19—H19*C*···O2	0.96	2.39	3.016 (2)	122
O4—H4···O3	0.83 (3)	2.06 (3)	2.629 (3)	126 (2)
O2—H2···O1^i^	0.82	2.11	2.9192 (18)	169

Symmetry code: (i) *x*+1, *y*, *z*.
