# Supplementary figures and images for: Crystal structure of hydro­cortisone 17-butyrate
Source: Acta Crystallogr Sect E Struct Rep Online. 2014 Nov 5;70(Pt 12):o1239–40. doi: 10.1107/S1600536814023903 (PMC4257404; doi:10.1107/S1600536814023903)

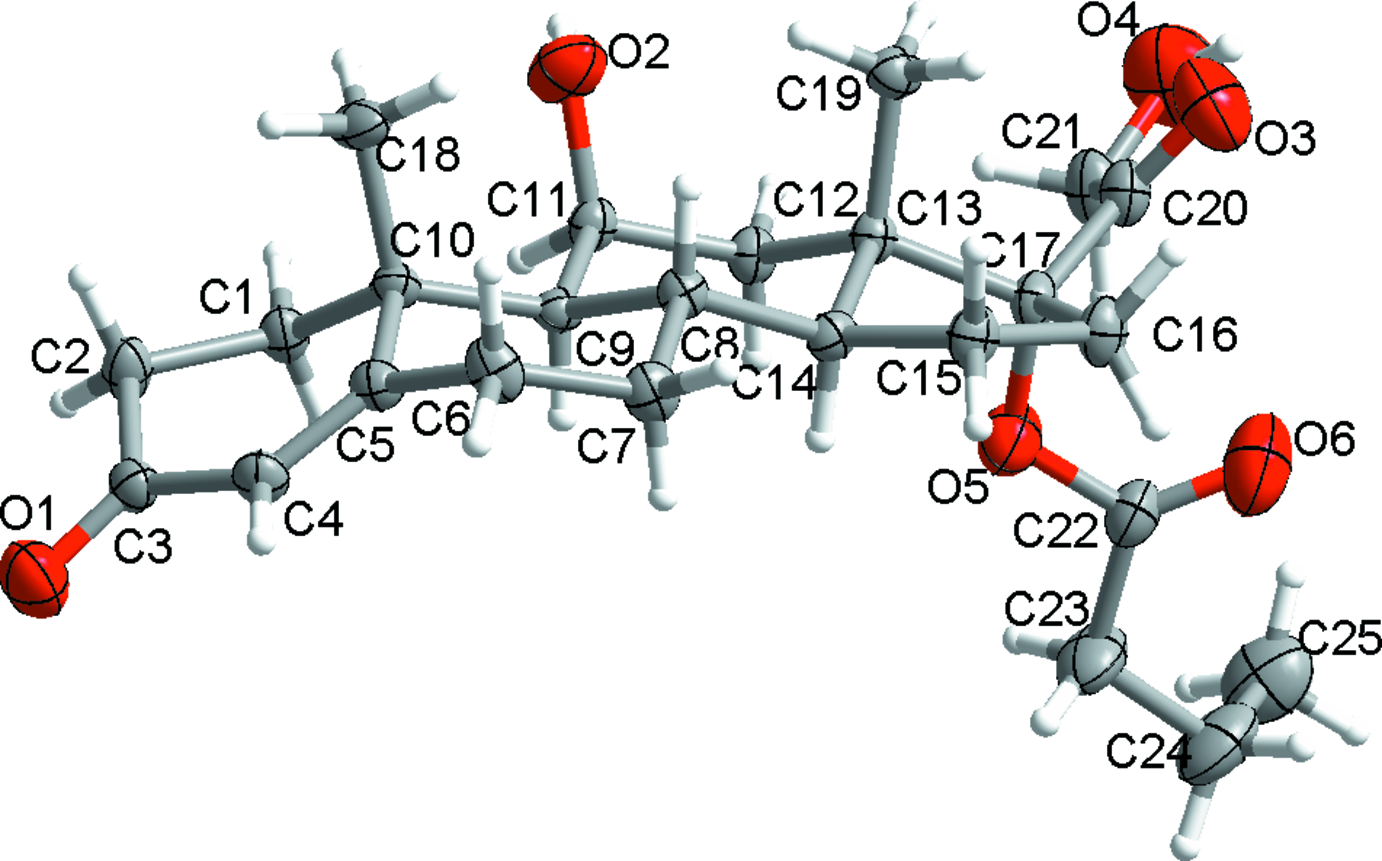

Supplement: Supplementary file 4 [file e-70-o1239-fig1.tif]
